# Relative Effectiveness of Mating Success and Sperm Competition at Eliminating Deleterious Mutations in *Drosophila melanogaster*

**DOI:** 10.1371/journal.pone.0037351

**Published:** 2012-05-25

**Authors:** Sean C. A. Clark, Nathaniel P. Sharp, Locke Rowe, Aneil F. Agrawal

**Affiliations:** Department of Ecology and Evolutionary Biology, University of Toronto, Toronto, Ontario, Canada; UC Santa Barbara, United States of America

## Abstract

Condition-dependence theory predicts that sexual selection will facilitate adaptation by selecting against deleterious mutations that affect the expression of sexually selected traits indirectly via condition. Recent empirical studies have provided support for this prediction; however, their results do not elucidate the relative effects of pre- and postcopulatory sexual selection on deleterious mutations. We used the *Drosophila melanogaster* model system to discern the relative contributions of pre- and postcopulatory processes to selection against deleterious mutations. To assess second-male ejaculate competition success (P2; measured as the proportion of offspring attributable to the experimental male) and mating success, mutant and wild-type male *D. melanogaster* were given the opportunity to mate with females that were previously mated to a standard competitor male. This process was repeated for males subjected to a diet quality manipulation to test for effects of environmentally-manipulated condition on P2 and mating success. While none of the tested mutations affected P2, there was a clear effect of condition. Conversely, several of the mutations affected mating success, while condition showed no effect. Our results suggest that precopulatory selection may be more effective than postcopulatory selection at removing deleterious mutations. The opposite result obtained for our diet manipulation points to an interesting discrepancy between environmental and genetic manipulations of condition, which may be explained by the multidimensionality of condition. Establishing whether the various stages of sexual selection affect deleterious mutations differently, and to what extent, remains an important issue to resolve.

## Introduction

It is intuitive to think of natural selection as a force operating to remove deleterious mutations from a population, although it is perhaps less conventional to think of sexual selection in the same context. However, if male siring success is condition-dependent, then natural and sexual selection may operate in the same direction on most genes [1]. Accordingly, condition-dependence theory predicts that sexual selection will facilitate adaptation through various mechanisms, including increasing the spread of beneficial alleles [2], and reducing the genetic load [2], [3], [4]. Likewise, condition-dependent sexual selection is predicted to enhance the rate and extent of adaptation in temporally fluctuating environments [5]. Indeed, several recent studies have demonstrated the efficacy of sexual selection against deleterious mutations [6], [7], [8], and the potential for sexual selection to reduce mutation load through selection on males [9], [10]. These effects may have occurred through precopulatory (e.g., mate choice) or postcopulatory (e.g., ejaculate competition) sexual selection, yet these studies did not attempt to partition total sexual selection amongst the components of siring success. Consequently, the relative extent to which selection against deleterious mutations occurs through pre- and postcopulatory processes remains unclear.

In *Drosophila melanogaster*, postcopulatory sexual selection is generated through differences in sperm number and ejaculate quality. For example, males produce seminal fluid proteins that have substantial effects on sperm transfer, sperm storage, female receptivity, ovulation, and oogenesis [11]. If ejaculate competition is condition-dependent, then most mutations should indirectly affect postcopulatory success via their effects on condition; there is some evidence that this is the case [12], [13].

Adaptations to sperm competition can be grouped into offensive and defensive categories. Sperm competition defence occurs when a male is the first to mate with a female (and thus he has to defend against potential future males' ejaculates), and can include such adaptations as strategic ejaculation of sperm and seminal fluids, mating plugs to retain his and block competitors' sperm, and behavioural guarding to inhibit females from remating [14]. Sperm competition offence occurs when a male copulates with a previously-mated female, and can include adaptations such as sperm displacement and strategic ejaculation [14]. For our experiment, we focused on sperm competition offence, which has particular importance to male *D. melanogaster* sexually selected fitness [15] and can be quantified as the proportion of offspring sired by the second male to mate a female (P2). We first performed a diet manipulation to establish whether P2 success was condition-dependent. We then compared P2 scores for a variety of mutant and wild-type genotypes. Our assay also provided a measure of pre-mating selection against these genotypes, allowing us to compare pre- and postcopulatory selection. Our objective was to determine whether components of pre- and postcopulatory sexual selection, specifically mate choice and sperm competition offence, could produce complementary effects on the mutation load in *D. melanogaster*. We predicted that mutant males would demonstrate reduced pre- and postcopulatory reproductive success relative to their wild-type competitors.

## Methods

### Study Organisms

Flies for the experiment were derived from a wild-type (+/+) outbred population of *D. melanogaster* originally collected in 1970 from Dahomey (now Benin), West Africa. The wild-type (+/+) Dahomey stock was maintained in the current laboratory for over six years at a population size of several thousand adults. We obtained six dominant deleterious mutant (M*_i_*/+; where *i* represents a given dominant mutation) stocks from the Bloomington *Drosophila* Stock Center (http://flystocks.bio.indiana.edu/), and each mutation was separately introgressed into the Dahomey background through at least ten generations of serial backcrossing. Each of these mutations affected separate autosomal loci, and was located on either the second (*Adv, Gla*) or third (*Dr, Gl, Ly, Sb*) chromosome. Mutant alleles had visible phenotypic effects on the eyes (*Dr*: eyes appear as vertical slits; *Gl*: eyes appear glossy and slightly reduced; *Gla*: eyes appear glossy), wings (*Adv*: wings contain an additional vein, *Ly*: wings have a reduced, rectangular appearance), or bristles (*Sb*: bristles are reduced to an abbreviated length); these visible markers allowed for easy identification of mutant individuals when scoring sperm competitor fitness. Four of these mutations (*Dr, Gla, Ly, Sb*) were chosen due to known viability selection, fecundity selection, and total sexual selection acting against them from an earlier experiment [8]; the other two (*Gl, Adv*) were chosen based on known viability effects [16]. All flies were cultured at 25°C under ∼60% relative humidity, on a 12L∶12D photoperiod.

### Mating Trial Design

Sperm competition offence was measured as the siring success of the focal male (either M*_i_*/+ or +/+) when he was the second male to mate with a female (i.e., the second-mating position). Focal males (M*_i_*/+ and +/+) for each of the six mutations tested were derived from a cross of +/+ females×M*_i_*/+ males. For each of the six mutations used in the experiment, a sample of experimental males was derived by crossing mutant males to outbred wild-type females from a common source population. Each mutation used in the experiment was introgressed into the Dahomey background (see Study Organisms); thus, a cross of mutant males by Dahomey females produced heterozygous mutant offspring and wild-type Dahomey offspring. Wild-type offspring produced from a given mutant cross were compared only with their mutant counterparts from that cross (i.e., the mutant and wild-type flies being contrasted always came from the same cross, and thus experienced identical juvenile competitive conditions). Using the *Gla* mutation as an example, *Gla* males were mated to +/+ females, and then *Gla* and +/+ male offspring from that cross were compared to each other to assess mutant/wild-type relative P2 and mating success for *Gla* only; thus, +/+ males derived from a cross of +/+ females×M*_i_*/+ males were statistically compared only with mutant males of the same cross. A standard fly stock homozygous for a recessive brown-eye mutation (*bw/bw*), which had been introgressed onto the Dahomey background, was used as the source of all females and all first mating-position (competitor) males in the experiment.

The design of the sperm competition experiment was standardised across each of the mutations tested. First-male matings were conducted at a rate of approximately 600 per mutation tested, and occurred *en masse* in shell vials (25×95 mm O.D.×height) containing standard yeast-sugar-agar medium by crossing virgin *bw/bw* males with virgin *bw/bw* females at a 10M∶10F sex ratio for a duration of 2 hours. This time window was chosen because it was sufficient for a single mating in *D. melanogaster*, and in previous mating experiments using fly lines of the Dahomey genetic background, only one mating pair was ever found to remate within the prescribed 2 hour observation window (SC pers. comm.). The 2 hour mating window is also conventional in sperm competition research in *D. melanogaster* (e.g., [17]). As the *bw/bw* mutation was recessive, all offspring of the first male carried the brown-eye phenotypic marker. Following the first-male mating, males were discarded, and females were retained individually in holding vials containing standard medium to ensure they had mated. Mating was confirmed by assaying for the presence of larvae in the holding vials 48 hours post-mating; all females that did not produce larvae were discarded. Three days after the first-male mating, mated females were allowed a three-hour window to remate with either a M*_i_*/+ or +/+ virgin second male (approximately five days old; individual matings were established at a ratio of approximately 3 M*_i_*/+ to 2 +/+ trials, as the likelihood of remating was predicted to be lower for M*_i_*/+ flies. Specific ratios: *Adv*: 299∶199; *Dr*: 300∶196; *Gl*: 236∶157; *Ly*: 224∶152; *Sb*: 286∶170; *Gla*: 292∶163). Offspring from this second cross were heterozygous with respect to the brown-eye allele; thus, these offspring were phenotypically wild-type with respect to eye colour, which distinguished them from the *bw/bw* offspring sired by the first male. Following the second-male mating, all males were discarded, and females were transferred into individual laying vials (vial 1) containing standard yeast medium. Females were kept in these vials for 48 hours, and then were transferred to a second set of laying vials (vial 2) for a period of 72 hours, after which females were discarded. Given that second-male copulations were not observed directly, mating was assumed to have taken place if there were wild-type or mutant offspring produced from the second-male mating. This is a reasonably accurate method of determining whether a female mated with the second male, given the strong last-male sperm precedence in this species [15]. All data from vials where females did not remate were removed from the dataset prior to P2 analysis; however, these data were useful in providing a measure of precopulatory selection (as described below).

### Postcopulatory Sexual Selection – Sperm Competition Offence

Sperm competition offence was assayed as the proportion of offspring sired by the second male. This proportion takes the form of *P2 = N_2_/(N_bw/bw_+N_2_)*, where *N_bw/bw_* is the number of offspring attributed to the *bw/bw* competitor male, and *N_2_* is the number of offspring attributed to the focal (M*_i_*/+ or +/+) male (three types of offspring were possible from the M*_i_*/+ crosses: *bw/bw*, M*_i_*/*bw*, and +/*bw*; just two types of offspring were possible from the +/+ crosses: *bw/bw* and +/*bw*). Offspring from laying vials 1 and 2 were counted and scored based on phenotype (wild-type, brown-eyes, or dominant mutation) on days 13 and 15 of their life cycle (exception: for mutations *Gla and Sb*, part of dataset was scored on days 12 and 14). To account for viability effects of the M*_i_* alleles, the M*_i_*/*bw* offspring count was omitted from the P2 calculation. Instead, the number of offspring sired by the mutant male was assessed as 2•*(N_+/bw_)* because the expected ratio of M*_i_*/*bw* to +/*bw* offspring is 1∶1 under conditions of no viability selection against the M*_i_* allele.

### Precopulatory Sexual Selection – Mating Success

While not the primary focus of this experiment, precopulatory sexual selection was approximated for each mutation tested by comparing the proportion of M*_i_*/+ and +/+ males that successfully mated with females during the second mating opportunity provided to each female. The second mating was considered successful if mutant and/or wild-type offspring were present among the progeny produced by a given female in vials 1 and 2. If only *bw/bw* offspring were produced (representing offspring of the first mating), then it was assumed that second males did not mate females. Our experimental design consisted of a “no-choice trial” using non-virgin females, in which each female was assigned a single male (M*_i_*/+ or +/+) as a potential mate; it was not possible for females to simultaneously choose between male types in this experiment.

### Condition Dependence

For each of the above assays (P2, mating success), we created high- and low condition males through a diet resource manipulation. Eggs laid by Dahomey females were transferred to vials in groups of 50 to standardise larval density and competition across condition treatments. High condition males were reared on standard medium, while low condition flies were reared on medium containing one-quarter of the standard volume of yeast and sugar. All P2 and mating success assay protocols for high and low condition flies were identical to those aforementioned for M*_i_*/+ and +/+ flies. Accordingly, individual matings were established at a ratio of approximately 3 low condition to 2 high condition trials (specific ratio: 431∶281), as it was predicted that, like mutant males, mating success would be lower for low condition males.

### Effects of Condition and Mutation on Body Size

The impact on male body size (an index of condition) of each of the six deleterious mutations, and our diet manipulation, was assayed for a group of virgin males separate from those used in the aforementioned fitness assays. Mutant and high/low condition males were produced and housed prior to weighing according to the same protocol as aforementioned for fitness assays. Flies were dried in an oven at 65°C for 25–27 hours, and then were weighed individually on a Sartorius microbalance.

### Analysis

All P2 data were analysed in R (v. 2.9.0; [18]) using general linear models with quasibinomial error structure, independently for each mutation and its paired wild-type competitor. Remating rate and body mass comparisons were assessed in JMP (v. 8.0.1) using a Chi-squared analysis and Student's t-tests, respectively.

## Results

### Sperm Competition Offence

**Figure 1:**
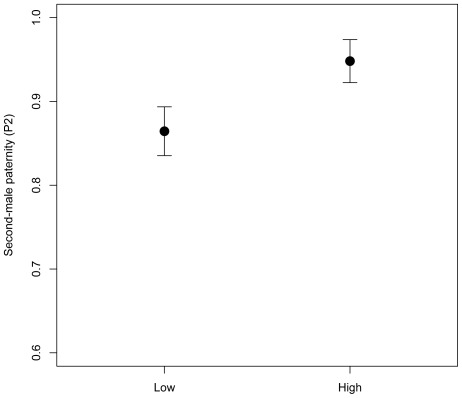
Relative offensive sperm competition success, given as second-male paternity (P2; mean +/− SE), for high- and low condition males. Sperm competition success was measured as the proportion of offspring produced by high- or low condition males relative to a standard competitor mated to a given female. High condition males showed significantly higher P2 relative to low condition males.

**Figure 2:**
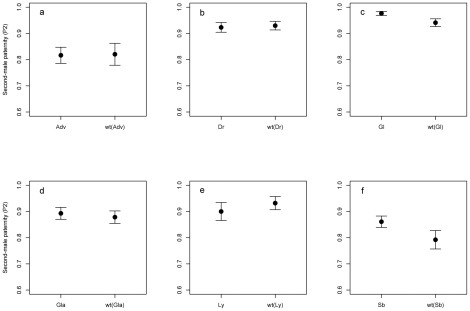
Relative offensive sperm competition success, given as second-male paternity (P2; mean +/− SE), for mutant and wild-type males (a. *Adv*; b. *Dr*; c. *Gl*; d. *Gla*; e. *Ly*; f. *Sb*). Wild-type treatments are listed as wt(M*_i_*), where M*_i_* represents the paired mutant treatment. Sperm competition success was measured as in Fig. 1. There were no significant differences between mutants and wild-types for any of the paired comparisons.

P2 was condition-dependent, but we could not detect an effect of the mutations on P2. Wild-type high condition males produced significantly more offspring than wild-type low condition males in the P2 mating position (*t*_1,83_ = −2.03, p = 0.0452; Fig. 1). However, none of the six mutations tested had a significant depressing effect on P2 (*Adv*: *t*_1,107_ = 0.339, p = 0.736; *Dr*: *t*_1,128_ = 0.192, p = 0.848; *Gl*: *t*_1,39_ = −1.22, p = 0.229; *Gla*: *t*_1,113_ = −0.576, p = 0.566; *Ly*: *t*_1,69_ = −0.262, p = 0.794; *Sb*: *t*_1,153_ = −1.62, p = 0.108; Fig. 2a–f).

### Mating Success

**Figure 3:**
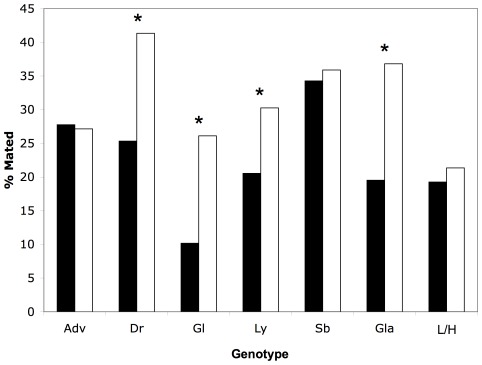
Relative mating success of mutant and wild-type males. Mating was deemed successful when mutant or wild-type males achieved copulation with a nonvirgin female; unsuccessful matings occurred when males failed to copulate. Black bars represent mutant (or low condition, L) males; white bars depict wild-type (or high condition, H) males. Mutant-wild-type pairs are presented according to mutant genotype. Significant differences are indicated by an asterisk.

Wild-type high condition males performed equally well to wild-type low condition males in obtaining a mating from once-mated females (*X* ^2^_1,655_ = 0.893, p = 0.345; Fig. 3). Reduced success of mutant males in obtaining a mating with once-mated females was shown for four mutations (*Dr*: *X* ^2^_1,496_ = 14.0, p = 2.0×10^−4^; *Gl*: *X* ^2^_1,393_ = 17.4, p<1.0×10^−4^; *Ly*: *X* ^2^_1,376_ = 4.64, p = 0.0313; *Gla*: *X* ^2^_1,455_ = 16.4, p<1.0×10^−4^; Fig. 3). For the remaining two mutations, mutant males were not significantly different than wild-types (*Adv*: *X* ^2^_1,498_ = 0.023, p = 0.879 and *Sb*: *X* ^2^_1,456_ = 0.123, p = 0.726; Fig. 3).

### Effects of condition and mutation on body size

As expected, high condition males were significantly larger in body mass relative to low condition males (18.3%, *t*_92.9_ = −9.04, p<0.0001; Table 1). Mutational effects on body mass varied, with half of the mutations significantly decreasing body mass (*Adv*: *t*_83.3_ = 4.23, p<0.0001; *Gla*: *t*_81.5_ = 4.41, p<0.0001; *Ly*: *t*_87.5_ = 6.68, p<0.0001; Table 1), and the other half showing no effect (*Dr*: *t*_92.7_ = −0.224, p = 0.823; *Gl*: *t*_82.3_ = 1.29, p = 0.199; *Sb*: *t*_91.3_ = 0.319, p = 0.751; Table 1). Pooling data for mutations that had a significant effect on body mass, wild-type flies were 12.0% larger than mutants (*t*_262.3_ = 8.35, p<0.0001), an effect size only two-thirds that of condition (Table 1).

**Table 1:** Effects of condition and mutations on body mass (mean +/− SE).

Gene	Mutation	Mass (mg) ± SE	% Difference	p-value
Condition	Low	0.199±2.91×10^−3^	18.3	**p<0.0001**
	High	0.236±2.81×10^−3^		
*Adv*	M	0.216±2.82×10^−3^	9.73	**p<0.0001**
	wt	0.237±4.11×10^−3^		
*Dr*	M	0.236±2.66×10^−3^	0.365	p = 0.823
	wt	0.235±2.78×10^−3^		
*Gl*	M	0.234±2.58×10^−3^	2.50	p = 0.199
	wt	0.239±3.71×10^−3^		
*Gla*	M	0.220±3.38×10^−3^	12.0	**p<0.0001**
	wt	0.246±4.96×10^−3^		
*Ly*	M	0.204±3.02×10^−3^	15.1	**p<0.0001**
	wt	0.235±3.48×10^−3^		
*Sb*	M	0.247±3.61×10^−3^	0.688	p = 0.751
	wt	0.249±3.94×10^−3^		

Gene corresponds to the locus of the dominant mutation, whereas mutation corresponds to whether the individual was wild-type or mutant with respect to that locus. Mass is given in milligrams with corresponding standard error. Percent difference in mass for a given gene is expressed as the difference between wild-type (High Condition) and mutant (Low Condition) masses, divided by the mutant (Low Condition) mass. P-values for significant differences are boldfaced.

## Discussion

Work to date probing the effects of sexual selection on female fitness (measured as offspring production) has focused on sexual selection *in toto*, and has generated each of neutral [19], negative [20], and positive [21] fitness effects. However, studies focusing on total sexual selection on novel deleterious mutations have collectively demonstrated that sexual selection acts to reduce the mutation load [6], [7], [8]. That is, sexual selection on males helps to eliminate alleles that would be bad for either sex. We found that males of several mutations showed reduced mating success, but performed equally well in ejaculate competition relative to their wild-type competitors. For the mutations tested, our results suggest that precopulatory sexual selection is more effective than postcopulatory sexual selection at reducing the mutation load. This implies that most of the previously demonstrated total sexual selection against several mutations used in our study [8] occurred at the precopulatory phase, as the previous study [8] looked at total sexual selection, and we parsed sexual selection into pre- and postcopulatory components.

Given that our diet manipulation showed reduced P2 for low condition males, we predicted that mutant males would likewise demonstrate lower P2; however, this was not what we observed. There are several possible explanations for this discrepancy. First, “condition” is likely to be multi-dimensional (e.g., [22]), and environmental manipulations may alter different aspects of condition than do deleterious mutations. Accordingly, P2 success may be insensitive to those aspects of condition altered by deleterious mutations. Alternatively, the mutations used here may have only weakly affected condition, perhaps making their downstream effects on P2 undetectable. These mutations were known to affect viability and/or fecundity [8], [16]. However, where present, mutational effects on body size tended to be smaller than for diet manipulation (Table 1). Furthermore, even the three mutations that did have significant effects on body size (*Adv, Gla, Ly*) showed no evidence of affecting P2. In contrast to our results, a recent mutation accumulation study reported deleterious effects on both P1 and P2 [23]. Because that study examines the effects of a more representative set of natural mutations, it seems likely that post-copulatory selection does help reduce mutation load. However, our results indicate that the amount of selection occurring through P2 may be very small for some types of mutations.

The precopulatory effect of these mutations was consistent with previous studies of some of them [8]. One interpretation of the earlier results [8] was that sexual selection occurred on these mutations via their effects on condition. However, it has been argued that mutations with obvious visible effects, such as those used here, may affect male mating success directly, rather than indirectly through condition [6]. In our experiment, precopulatory success was unaffected by our diet-based manipulation of condition, and yet the mutations we tested still seemed to experience selection at the precopulatory stage. These observations suggest two things. First, the “no-choice” measure of precopulatory success used here (see Methods) might have been a rather blunt assay of true mating success, which may be insensitive to condition. Previous studies using very similar diet manipulations have shown that male mating success is strongly condition-dependent when assessed in competitive mating assays (e.g., [24]). Second, this observation further supports the notion that these mutations affect mating success directly, rather than indirectly via condition.

Our experiment represents a preliminary attempt to elucidate the relative importance of the different components of sexual selection against mutation load. Our study revealed interesting discrepancies between environmental and genetic manipulations, which may be resolved by using inbreeding or mutation accumulation to manipulate genetic quality (such manipulations are likely to be more representative of segregating mutations). Disentangling the effects of sexual selection on deleterious mutations into pre- and postcopulatory processes remains an unresolved challenge in understanding how and when sexual selection acts on deleterious mutations.
